# Regulatory strategies for rare diseases under current global regulatory statutes: a discussion with stakeholders

**DOI:** 10.1186/s13023-019-1017-5

**Published:** 2019-02-08

**Authors:** Andrew E. Mulberg, Christina Bucci-Rechtweg, Joseph Giuliano, David Jacoby, Franklin K. Johnson, Qing Liu, Deborah Marsden, Scott McGoohan, Robert Nelson, Nita Patel, Klaus Romero, Vikram Sinha, Sheela Sitaraman, John Spaltro, Vivian Kessler

**Affiliations:** 1 Amicus Therapeutics, Inc., 1 Cedar Brook Drive, Cranbury, NJ 08512 USA; 2 Novartis Pharmaceuticals Corporation, One Health Plaza, East Hanover, NJ 07936 USA; 3 BioMarin Pharmaceutical Inc., 770 Lindaro St, San Rafael, CA 94901 USA; 4 Ultragenyx Pharmaceutical, Inc., 60 Leveroni Ct, Novato, CA 94949 USA; 5 Vertex Pharmaceuticals, 50 Northern Ave, Boston, MA 02210 USA; 6 Johnson & Johnson, 920 Route 202 South, Raritan, NJ 08869 USA; 7 Critical Path Institute, 1730 East River Rd, Tucson, AZ 85718 USA; 8 Merck & Co, Inc. 351 North Sumneytown Pike, North Wales, PA 19454 USA

## Abstract

Rare or orphan diseases often are inherited and overwhelmingly affect children. Many of these diseases have no treatments, are incurable, and have a devastating impact on patients and their families. Regulatory standards for drug approval for rare diseases must ensure that patients receive safe and efficacious treatments. However, regulatory bodies have shown flexibility in applying these standards to drug development in rare diseases, given the unique challenges that hinder efficient and effective traditional clinical trials, including low patient numbers, limited understanding of disease pathology and progression, variability in disease presentation, and a lack of established endpoints.

To take steps toward improving rare disease clinical development strategies under current global regulatory statutes, Amicus Therapeutics, Inc. and BioNJ convened a 1-day meeting that included representatives from the Food and Drug Administration (FDA), biopharmaceutical industry, and not-for-profit agencies. The meeting focused on orphan diseases in pediatric and adult patients and was intended to identify potential strategies to overcome regulatory hurdles through open collaboration.

During this meeting, several strategies were identified to minimize the limitations associated with low patient numbers in rare diseases, including the use of natural history to generate historical control data in comparisons, simulations, and identifying inclusion/exclusion criteria and appropriate endpoints. Novel approaches to clinical trial design were discussed to minimize patient exposure to placebo and to reduce the numbers of patients and clinical trials needed for providing substantial evidence. Novel statistical analysis approaches were also discussed to address the inherent challenges of small patient numbers. Areas of urgent unmet need were identified, including the need to develop registries that protect patient identities, to establish close collaboration and communication between the sponsor and regulatory bodies to address methodological and statistical challenges, to collaborate in pre-competitive opportunities within multiple sponsors and in conjunction with academia and disease-specific patient advocacy groups for optimal data sharing, and to develop harmonized guidelines for data extrapolation from source to target pediatric populations. Ultimately, these innovations will help in solving many regulatory challenges in rare disease drug development and encourage the availability of new treatments for patients with rare diseases.

## Introduction

Rare or orphan diseases are defined in the United States as diseases and conditions that have an incidence of < 200,000 patients, or elsewhere in the world as having a prevalence ranging from < 1:2000–< 1:50,000 [1, 2]. Approximately 80% of the thousands of defined rare diseases have an underlying genetic basis and approximately three-fourths affect children [1]. Many of these rare diseases lack treatments or cures and are fatal, making new treatments potentially transformative for the lives of patients [1]. However, there are several unique challenges surrounding the development of orphan diseases treatments. Low patient numbers, an incomplete understanding of the disease pathology, phenotypic heterogeneity, and a lack of established endpoints are barriers to efficient and effective clinical trials [2–4], which can make meeting regulatory requirements for drug approval challenging.

An interactive, 1-day BioNJ meeting, “Developing Rare Disease Regulatory Strategy Under Current Global Statutes: A Stakeholder Discussion,” was held at Amicus Therapeutics, Inc. on March 28, 2018 to discuss challenges in developing rare-disease regulatory strategies under current global regulatory statutes. The meeting included representatives from the Food and Drug Administration (FDA), biopharmaceutical industry, and not-for-profit agencies, and focused on orphan diseases in pediatric and adult patients. It was attended by more than 90 leaders in various therapeutic areas, regulatory policy, pharmacology, biostatistics, and research ethics. This summary of the proceedings identifies potential strategies to overcome regulatory hurdles through open collaboration.

### Concept and design consideration in rare diseases: Clinical development plans

#### Challenges in designing clinical trials for orphan diseases

Statutory standards for approval of orphan drugs developed for treatment of rare diseases are the same as those of common diseases: there must be substantial safety and efficacy evidence from well-controlled trials [3, 5]. However, in some cases it may not be possible to meet these standards when developing orphan drugs [2–4]; therefore, the FDA applies scientific judgment and regulatory flexibility when making decisions about drug development and approval in rare diseases [4–6]. Many orphan diseases are serious and/or life-threatening and primarily affect pediatric patients, underscoring both the challenges and urgency of effective drug development [1]. The inappropriateness of administering some therapies to healthy controls and the rarity of orphan diseases also pose logistical challenges for conducting clinical trials [7–9].

#### Using historical control data

External historical control data describing the natural history of a rare disease play a major role in developing treatments for orphan diseases where performing a randomized, placebo-controlled trial may not be feasible or appropriate [5, 10]. For diseases with substantial heterogeneity in clinical presentation, improved predictive ability based on the natural history of the disease may inform inclusion/exclusion criteria to facilitate an effective clinical trial program and help identify potential biomarkers to guide treatment [4, 11]. In particular, natural history data can inform endpoint selection in the context of event rate and variability of disease presentation to ensure that the number of patients enrolled should allow sufficient power to detect efficacy [10].

One example demonstrating effective use of a historical control was in the development of cerliponase alfa enzyme replacement therapy (ERT) for the treatment of ceroid lipofuscinosis neuronal type 2 (CLN2), a late infantile form of Batten’s disease that generally presents between 2 and 4 years of age and leads to rapid progressive neurodegeneration and death [12]. The pivotal study for cerliponase alfa was developed through close collaboration and communication between the sponsor and regulatory authorities, during which several methodological and statistical concerns were sequentially raised and addressed. Initially, questions arose regarding the comparability of the sample populations. The presentation of CLN2 is not highly variable, but underlying differences in co-variables, such as age, sex, disease alleles, and baseline scores, may have existed between the treated population and the natural history cohort [13]. To address this concern, matching methodologies were incorporated, including adjustment for co-variables and use of many-to-one matching to compare one study subject with multiple historical controls; following these adaptations, all analyses consistently demonstrated a significant effect of cerliponase alfa ERT [13]. Additional concerns were raised regarding the comparability of rating assessments for the treated population and the natural history cohort; the sponsor made changes to ensure comparability, including training of assessment scale raters outside of the study to ensure similar interpretation, establishing clear anchor point definitions (ie, what constitutes a meaningful change in score), and using well-accepted comparative methodology for video/live assessments and assessment scales when versions differ between the historical control group and the study population. The process consisted of an iterative review of the data, and hurdles were overcome by collecting data in a verifiable manner and identifying methodological/statistical approaches to eliminate potential design/analysis flaws. Ultimately, the issues raised during regulatory review were mutually instructive for both parties, and the pathway to approval was facilitated by frequent communication and collaborative adaptation of study methodology and statistical approaches. Recommendations and considerations for use of external historical controls are discussed in the 2000 International Conference on Harmonisation (ICH) E10 Choice of Control Group guidance [14]. The cerliponase alfa ERT clinical development program provides a real-life example for improving comparability between an external historical control group and the population under study (ie, what a concurrent control group may have looked like).

#### Modeling disease progression from natural history data

Disease progression can be modelled based on natural history data, which can be used as part of clinical trial simulations [15]. Simulating disease progression for a defined patient population can be used to evaluate, inform, and optimize clinical trial design, for example by projecting required sample sizes, identifying relevant patient populations, estimating the magnitude of treatment effect, and defining the required duration of follow up [15]. Quantitative modelling of disease progression increases our understanding of how biomarkers and other relevant sources of variability can be used as surrogate markers of disease progression [11]. For example, a robust quantitative understanding of disease progression may be used to define cut-off points for clinical trial enrollment, although this may not be necessary if the full distribution of a quantitative marker can be modeled as a continuous variable across a population [4]. In the case of Duchenne muscular dystrophy, forced vital capacity is being investigated as a potential marker for disease progression in the context of potential covariates, such as anthropomorphic measurements, baseline severity, and steroid use. Accordingly, quantitative markers may help define a specific context for drug use, facilitating efficient clinical trial design and drug development, and streamlining the pathway towards regulatory acceptance [3].

When using natural history databases, it is essential that they are as current as possible and continuously updated with new data. However, creating and updating the databases is time consuming, and since detailed data collection requires significant time and resources, financing these efforts can be an obstacle. In addition, enrolling patients in registries and other real-world studies is an ongoing challenge; therefore, novel methods of collecting natural history data are needed, and initiatives to facilitate and encourage data sharing, such as making individual patient data available to qualified researchers, need to be considered. The Yale Open Data Access (YODA) project is one such initiative, through which data holders can share their clinical research data responsibly and researchers can request access to clinical trial data [16]. Although data sharing is beneficial to the research community, it can also deter a patient from providing consent to join a registry. Development of nonproprietary disease-based registries should be encouraged and facilitated in conjunction with patient advocacy groups. Likewise, innovative study designs and collaboration between stakeholders outside commercial therapy development offer an option for efficiently generating data sets on patient natural history and improving the accuracy of simulations [4, 5, 7, 9]. It is notable that unless designed prospectively to be used as a comparator for clinical trials, both historical cohorts and registries have the same limitation; they may not include data on the endpoints used in clinical trials.

#### Modeling and simulation: Innovation in clinical trial design

Good examples of innovative approaches in clinical trial design abound. With laronidase for mucopolysaccharidosis I, data was used across several studies to determine exposure and inform dose selection. The phase 3 study used a novel composite clinical endpoint with defined clinically significant thresholds for each component to evaluate treatment effect and identify treatment responders [17]. Drug exposure-response analyses have also been used to extrapolate data from adult populations to pediatrics; an example of this is in the extrapolation of exposure-response data for infliximab from adults with ulcerative colitis to pediatric patients, where the exposure-response relationship during the induction phase did not appear to be different. However, data were limited for evaluating the maintenance phase, and in the end, it may have been beneficial to perform a dose-ranging study [18]. Simulation/modeling approaches can also be used to reduce the number of clinical trials; such an approach was used in the evaluation of eliglustat for Gaucher’s Type I disease, in which drug-drug interactions were expected based on its metabolism by CYP2D6 and CYP3A. Therefore, simulations using physiologically based pharmacokinetic modelling were used to predict changes in exposure with various CYP inhibitors, which informed the prescribing information and eliminated the need for further clinical trials [19].

Another example of innovation in clinical trial design is the ‘blind start’ approach—a novel, 4-treatment sequence, double-blind, placebo-controlled cross-over study design that can be applied to rare diseases [20]. All patients receive a minimum duration of active treatment (e.g., 24 weeks, depending on the anticipated duration of treatment required to demonstrate an effect); however, patients are randomly assigned to begin active treatment at different predefined timepoints (at randomization or following 8, 16, or 24 weeks of placebo treatment in 3 of the 4 sequences) [20]. This study design offers an estimated treatment effect size similar to that of a randomized parallel-group study, and its power to detect a real effect on clinical endpoints is better than that of a traditional parallel study design with the same sample size [20]. This study type maintains the ability to provide objective assessment of placebo-controlled data despite having low patient numbers, while also ensuring that all patients receive the investigational treatment [20]. This study design was used in the phase 3 pivotal study of the recombinant human β-glucuronidase ERT (vestronidase alfa) [21], which is approved for the treatment of patients with the ultra-rare disease mucopolysaccharidosis VII.

#### Collaboration

Collaboration can also improve rare disease clinical research. The Critical Path Institute (C-Path), for example, is a nonprofit, public-private partnership with FDA created under the auspices of the FDA’s Critical Path Initiative program in 2005, which is working with industry, government, academia, and advocacy groups on several initiatives to support development of novel therapies [22]. This neutral third party works with stakeholders to overcome challenges to effective drug development, including data access, anonymization of patient health information, and enforcement of data use agreements [22]. In conjunction with the FDA and the International Society of Pharmacometrics, C-Path also initiated discussions surrounding the need for improved modeling and simulation tools; these efforts paved the path to regulatory approval of publicly available, fit-for-purpose, quantitative drug development tools for drug development programs [23]. Educating and engaging healthcare professionals, patients, and patient organizations is also imperative because the effectiveness of broad research initiatives, such as non-proprietary registries, requires adequate stakeholder support.

### Registry development

Rare disease registry development presents three main challenges: data collection using standardized language, data anonymization, and data accessibility [24]. To facilitate the collection of complete, meaningful information from patient registries, it is important to have a defined minimal data set and support to collect these data in all enrolled patients. With regard to data anonymization, legal and regulatory requirements in different jurisdictions are varied and dynamic, so it is important for investigators to understand the privacy requirements surrounding real-world data collation; patients with rare diseases enrolled in registries have a higher risk of being identifiable, despite data anonymization [1, 7]. Adequate controls need to be in place to ensure appropriate data use and confidentiality. Methods for addressing these challenges are described below.

#### Data anonymization

Given the low prevalence of rare diseases, it is paramount to ensure that data collected in registries does not reveal the identity of a patient and their involvement in a research study [24]. Therefore, best practices for reducing the risk of revealing a patient’s identity need to be developed and applied. These include providing data contributors with a clear description of applicable regulations (country- and region-level) and a comprehensive guide on how to anonymize variables to reduce patient ‘distinguishability’ to levels that are internationally compliant and are suitable for cross-border data transfer (e.g., eliminating Social Security number or other unique identifiers and converting dates to timeframes). There are several statistical and scientific methodologies that can minimize the risk for individual patient-level data inadvertently identifying a patient. From a study sponsor’s perspective, rules should also be established for anonymizing the sponsor’s name on registry records, as well as study identifiers and any drugs being evaluated using processes that are governed by formal, comprehensive Data Contribution Agreements (DCAs) and Data Use Agreements (DUAs).

#### Data accessibility

All registries must be developed with data accessibility parameters in mind, specifically which parties can access data and to what extent (e.g., full or limited access to data sets for specific patients or specific data for each patient) [10]. Likewise, data portability should be considered, for example, limiting access to data via a registry portal or determining whether data can be independently transported and shared.

DCAs may be implemented as a legal framework to govern the data-sharing process among established collaborators. These frameworks allow data contributors to define parameters for data sharing (access by whom and to what extent) or to institute moratoria on level of data sharing (e.g., until drug approval, primary analysis completion/publication, or a fixed date).

Alternatively, a set of Terms and Conditions or DUAs can be implemented. These agreements can be used to help define who can access the data, for example, by formally stating the criteria required to meet the definition of a “qualified researcher” and establishing and communicating policies for the submission and review of data access requests. To establish parameters for how the data may be accessed and used (e.g., data transport, only remote views, redistribution), a contextualized comprehensive analysis of advantages and disadvantages of each approach should be performed that considers the purpose of the registry. Although data redistribution should generally be avoided, any provisions for redistribution should be clearly indicated in the registry Terms and Conditions/DUA and enforced accordingly. In addition, all DUAs should include provisions for protecting patient identities and confidentiality, as well as publication rights, with appropriate attribution.

### Dose selection and dose ranging

Establishing an understanding of dose-response relationships to inform dose selection in rare disease is challenging for a variety of reasons, particularly in pediatric populations. The patient populations are small, limiting the use of extensive dose-ranging phase 2 studies. In addition, patients are often in relatively poor physical condition, which can limit the number and type of procedures that can be performed. In pediatric patients, blood sample collection is particularly challenging because of lower blood volumes, which limit regular/redundant blood sampling, and ethical considerations limit the use of biopsy approaches. Moreover, it may only be possible to test one dose, further limiting assessment of exposure-or dose-response relationships.

Knowledge of the chemistry, formulation, and toxicology of a drug is highly valuable during development for use in orphan diseases, including in pediatric populations [15, 25]. Treatment effect is generally related to drug concentration, so extrapolating pharmacology and toxicology data (e.g., by evaluating the relationship between drug concentration and biomarkers) may provide an efficient approach to selecting doses to be used in registration studies, including fixed doses due to pharmacokinetic variability [15]. In particular, data relating to exposure-response relationships, which may be further informed by non-clinical studies, may aid dose selection for studies in patients with orphan diseases and help avoid adverse events and drug interactions, especially when the course of a disease and response to treatment is expected to be similar between adults and children [15]. Furthermore, prior knowledge of drug-drug interactions and the feasibility of treating special populations, such as patients with renal or hepatic insufficiency, can help reduce the number and complexity of clinical trials, facilitating a more efficient use of limited healthcare resources [25].

Another possible approach to informing dose selection for orphan drugs in rare diseases, where feasible, is the substitution of clinical endpoints with biomarkers, ideally in the form of a panel of biomarkers representing various aspects of the disease [11]. However, many rare diseases do not have sufficiently characterized biomarkers, and a better approach may be to focus on the totality of trends in clinical efficacy and safety data, utilizing the entire body of available evidence [15], followed by clinical trial exposure-response simulations and quantitative systems pharmacology (QSP) modeling if reliable biomarkers exist. QSP modeling uses a mechanistic approach, incorporating molecular drivers of the disease and effects at the cellular and organ levels, and can provide support for a given dose or for evaluating different dosing regimens [26]. In some situations, dosing may be defined largely based on safety assessments or even predicted toxicity according to toxicological studies. Other analytical approaches may also be necessary, such as in silico modeling of the dose-response relationship, although endpoints would ideally be compared against a parallel study arm receiving a placebo control [15]. Translational modeling from knock-out mouse models or other preclinical models may help to support the trends in efficacy and safety and in silico evaluations.

If enough clinical efficacy data are not generated as part of the dose-ranging process, mechanisms need to be considered for dose optimization once a proof of concept for treatment effect has been established. Adaptive study design, where the study design is continuously modified as more data are generated (e.g., after interim data are input into clinical simulations), is a practical method of dose-ranging and dose optimization for patients with rare diseases [5, 27, 28]. An adaptive approach can also ensure that patients are administered the most appropriate treatment and offers the flexibility of incorporating traditional phase 2 and 3 study designs into a single trial to efficiently investigate a new therapy in a small patient population [27].

Furthermore, although pharmacokinetic exposure or exposure-response relationships may be extrapolated from adults to various pediatric age groups, the feasibility of doing so should be assessed on a case-by-case basis, considering both the drug and patient population. Children may present with more severe forms of a disease compared with adults, limiting extrapolation. There also may be technical considerations when extrapolating efficacy or safety data from adults to inform dose selection in rare pediatric diseases (e.g., whether the same methods of measurement can be used in adults and children).

### The role of data extrapolation from varying age cohorts: Regulatory requirements for pediatric/rare disease drug development

Pediatric patients should have access to products that have been appropriately evaluated in pediatric populations, which means that product development programs for therapies that can reasonably be expected to be used by children should include adequate and well-controlled clinical trials in children, when appropriate, to meet the same evidentiary standards as studies in adults [3]. There are also potential benefits for sponsors who opt to investigate new therapies in children, including extended data exclusivity [2]. However, there are several ethical considerations pertaining to performing studies in children. Children should only be enrolled in clinical trials if the study objectives cannot be met by enrolling subjects who can provide informed consent (ie, adults). If children are included in a clinical trial, the risks to which they are exposed either must be low absent potential clinical benefit, or must present a reasonable balance of risk and potential clinical benefit [29].

Efficacy data for a drug may be extrapolated from a source to a target population (eg, from adults to pediatric populations or from adolescents to younger pediatric populations). Small but well-defined cohorts in different age groups might be considered if extrapolation into older children or adults with milder forms of the disease is of interest. Ultimately, data extrapolation must be justifiable [29]. Data can be extrapolated in cases where the disease course and response to therapy is sufficiently similar between the source and the pediatric target population, for example when there is evidence of comparable disease pathogenesis, criteria for disease diagnosis and classification, measures of disease progression, and pathophysiologic, histopathologic, and pathobiologic characteristics across populations [25, 29–31]. Likewise, it is necessary to have a sufficient understanding of how a target pediatric population may resemble (or differ from) a reference population in terms of disease pathophysiology, possible biomarkers and study endpoints, physiology, alternate treatment options, and any potential pharmacological differences. It should be noted that dosing and safety data may not be fully extrapolated, although this does not mean that data from sources other than pediatric populations cannot be leveraged [15].

Drug approval rates in pediatric populations have been hindered by low disease prevalence, heterogeneous populations, low event rates, lack of standardized study design (including study endpoints), and variability in standard of care [11]. The FDA’s willingness to accept extrapolated data to support a new therapeutic approval has been tempered by instances of failures of data extrapolation [32]. Examples of this include failures because single well-controlled studies were thought to be sufficient but later proved to be an inaccurate representation of the true treatment effect in children, or because exposure-response relationships cannot be identified in the overall pediatric population or in subgroups [32]. Increasingly, studies that are difficult to perform in pediatric populations are being requested or required by the FDA.

Ultimately, the foundation of data extrapolation in pediatric populations is dependent on the accuracy of the assumptions that are made and the quality and quantity of data, including in cases where data are used in simulations or innovative statistical approaches, such as Bayesian statistics [10, 15, 25, 28]. Assumptions must be justified using scientific processes to minimize uncertainty and should be prospectively identified and managed. Potential differences between the target and source populations can be quantified using mechanistic or empiric approaches [31], with the former relying on data that support similarities or differences and the latter relying on establishment of mathematical formula or models to do so. Furthermore, any assumptions may need to be revisited and updated as more data are generated. For example, confirmatory data may be required following approvals based on data extrapolation, which may result in either an expansion or narrowing of a drug’s indication as more data on the clinical effectiveness and use of a drug are available from postmarketing studies [5, 10]. In principle, data may be extrapolated from one indication to another in cases where both indications have the same molecular target. However, even as the same molecular pathology may underlie multiple diseases, differences in tissues and cell types, compensatory/resistance mechanisms, and clinical trial endpoints can complicate data extrapolation.

Methodologies and strategies for extrapolating data to pediatric populations need to be harmonized across regulatory agencies globally to improve the speed of access to new therapies for pediatric patients, while also limiting the number of children that are exposed to investigational therapies during clinical trials supporting regulatory approval [8, 33]. Future guidelines from the International Conference on Harmonization of Technical Requirements for Pharmaceuticals for Human Use (ICH) are expected to address and align the terminology surrounding data extrapolation for pediatric populations and discuss how a systematic approach may be applied. Furthermore, guidance on potential study designs and statistical approaches when incorporating extrapolated data into pediatric drug development plans may be provided [33].

Accordingly, before extrapolating data to pediatric populations in rare diseases it is important to identify all relevant data to minimize uncertainty about its applicability [29]. Data can be derived, for example, from formal clinical trials, real-world evidence (RWE), and non-clinical studies. Relevant data that can be extrapolated should also be identified early and in collaboration with relevant regulatory authorities, ideally as part of a pediatric investigation plan that is conceived at the time of initiating studies in adult populations [15]. As the science of data extrapolation between populations is advanced and experience is gained, it is hoped that an expanded and globally standardized approach will be developed [29, 33].

### Patient population and endpoint selection

As the level of confidence increases regarding the similarity of the disease characteristics and response to therapy between adult and pediatric populations, the required level of evidence from pediatric populations to achieve marketing approval for a drug in a pediatric population decreases. Approximately 60% of pediatric programs require at least one adequate, well-controlled efficacy trial to be performed (either no extrapolation or partial extrapolation used) before marketing approval is granted [32, 34]. However, data to support an application for approval in a pediatric population may also be generated using studies powered on a surrogate endpoint, controlled studies without formal statistical power, non-controlled descriptive efficacy/safety studies, small dose-ranging studies, small pharmacokinetic (PK)/pharmacodynamic studies (single–dose-level matching adult exposure) or PK/safety only [32]. The extent, nature, and combination of studies required depends largely on the specific drug under study and the proposed indication.

Challenges and unique approaches also exist in the development of targeted therapies for low-frequency molecular subsets of a disease where the drug is likely to be efficacious [35]. For example, some patient subsets are too small to evaluate as part of a clinical trial. In these cases, it may be possible to group putatively similar molecular subsets in a single trial or to enroll all patients with the clinically-defined disease to allow evaluation of treatment efficacy across molecular alterations. When grouping patients with different molecular alterations, there must be clear support for a similar pharmacological response to treatment from clinical or nonclinical drug studies, in silico or mechanism-based evidence, or other sources. An initial molecular subset may also provide proof-of-concept that can be built upon as more data become available [5, 35]. For example, the initial marketing approval for ivacaftor for children with cystic fibrosis, a condition that has a well-understood natural history and underlying pathology, was limited to 10 genotypes, but the approval was sequentially expanded to include 38 genotypes as clinical and laboratory studies progressively identified both responsive and non-responsive genotypes.

Likewise, appropriate endpoints need to be chosen for a pediatric population; including endpoints that are relevant to a pediatric population in adult studies is one method of streamlining a future development program for a pediatric population. There is also a need for development of new endpoints that are more sensitive and reproducible, which may be accomplished using registry data. In addition, collation of RWE, for example via registries or patient access programs, can generate an expanded evidence base that may be extrapolated to a pediatric population [36]. These developments require close collaboration among all stakeholders, including patients, academia, companies, regulators, and payors [24, 36]. Such collaboration can reduce fragmentation within a therapy area, particularly among patient organizations, by ensuring a consistent approach and efficient use of resources [24, 36].

### Novel design and statistical considerations in rare disease drug development

The small sample sizes associated with studies of rare diseases can restrict study design options, replication, and the use of inferential statistics, which means that novel and innovative statistical designs may need to be considered to assist in assessing the evidence of the efficacy and safety of a potential treatment [5]. Enrichment is one option, wherein patients are enrolled on the basis of a prospectively defined characteristic that is believed to improve the probability of detecting a treatment effect compared with an unselected patient population [37]. This can include defining a narrow patient population to reduce patient variability, selecting patients who have a higher probability of experiencing an endpoint, or selecting patients who are expected to be more likely to respond to treatment [37]. However, an enrichment design may reduce the generalizability of the results to an unselected patient population.

Incorporation of RWE for patients receiving a standard of care (SOC) as a control, in the form of an external historical or internal concurrent control, is also critically important for quantifying benefits or risks pertinent to patients as well as for increasing the probability of success [38]. A patient-centric approach focuses on quantifying treatment outcomes of a new medical product for each individual patient, either through traditional clinical evaluations or patient-reported outcomes. The endpoint for quantifying an individual treatment outcome can be a change from the baseline measure for studies in which patients’ disease conditions are relatively stable; conversely, in studies of patients with rapidly progressive disease, the endpoint may be the difference in post- and pre-treatment slopes.

These considerations, as well as the practicality of rare disease clinical trials in different diseases/patient populations, may lead to a unique design choice from a list of options, including randomized parallel (blinded or unblinded) group design and single-arm trial design with either internal or external natural history controls [39]. In addition to the traditional parallel-group design, variations such as a randomized delayed start (RDS) design or a randomized enrichment (RE) design with internal RWE control (RWE-RE design) can also be used. The RDS design, which is suitable for patients with relatively stable disease condition over the duration of the trial, consists of two stages: for stage 1, patients are randomized to receive a new treatment or a control; for stage 2, patients who received control in stage 1 switch to the new treatment. The primary analysis is based on an integrated analysis of efficacy that combines stage 1 inter-group and stage 2 intra-patient comparisons of treatment and control. The RWE RE-design also has two stages: the first stage is an open-label observational study of RWE of a SOC over a suitable duration to quantify disease progression; patients from stage 1 who meet outcome-driven enrichment criteria are randomly assigned to receive a new treatment or remain on the SOC. The primary endpoint may be based on difference in post- and pre-treatment slopes or some difference of observed and predicted outcome. Both the RDS design and RWE-RE design have received support from regulatory agencies for use in rare disease clinical trials.

Small sample size also poses substantial challenges for statistical analyses. A traditional two-sample test, which was not developed for medical research applications, assumes that investigators or trial sponsors are equally ignorant of effects both for an investigational treatment and a control. For most clinical investigations of the investigational treatment, the selection of the control is often based on substantial knowledge of RWE of SOC and thus a patient-centric approach to statistical analysis is an intra-patient analysis of treatment benefits. Such an analysis should be flexible enough to adjust for various potential sources of bias in the choice of the control, whether it is a natural history or concurrent control. This leads to an inter-group (EIG) analysis that is highly efficient, minimizing the number of patients required for enrollment, and very robust. The application of EIG analysis with various design options enables adequate and well-controlled studies that are 50 to 65% more efficient than traditional methods, making scientifically rigorous clinical studies for rare disease drug development feasible.

## Conclusions and recommendations

Based on the feedback and discussions from leaders in various therapeutic areas in the rare disease space and experts in regulatory policy, pharmacology, biostatistics, and bioethics during this meeting, several strategies were identified to streamline the clinical development process and facilitate regulatory approval of treatments for rare diseases. These strategies focused on mitigating the key barriers to drug development, including low patient numbers, a poor understanding of the mechanisms of disease pathology and progression, a lack of established clinical trial endpoints or surrogate markers, and variability in disease presentation, all of which hinder efficient treatment comparisons and statistical analyses in clinical trials.

Effective use of natural history data and generation of historical control data as external controls was identified as a key element in addressing several challenges in rare diseases, including small patient numbers, poor disease understanding, and a lack of established endpoints/biomarkers. These data serving as external controls potentially can be used in place of placebo comparators in clinical trials, thereby limiting the number of patients exposed to placebo. Adoption of these principles has been presented recently and propagated by the US FDA in recent guidance specifically focusing on development of gene therapy approaches for rare diseases. In addition, natural history data can be used in performing clinical trial simulations and in studying disease pathology and heterogeneity, which may help to identify study inclusion/exclusion criteria, potential biomarkers, and appropriate endpoints.

Several novel clinical trial designs/approaches were discussed to minimize patient exposure to placebo and to minimize the number of patients required for inclusion in clinical trials, which is particularly important for reducing the risks to pediatric patients:

- Blind-start crossover and randomized delayed start trials
- Single-arm trials with internal or external controls
- Trials incorporating real-world evidence controls
- Adaptive study designs that are modified as more data are collected
- Many-to-one matching methodologies, allowing comparison of one study subject with several historical controls
- Randomized enrichment approaches to define a narrower patient population, reducing variability and improve the probability of detecting a treatment effect
- Quantitative modeling of disease progression to identify potential biomarkers and surrogate endpoints
- Modeling and extrapolation approaches for dose finding (e.g., from pharmacology and toxicology data)
- Modeling approaches for extrapolation of data from source to target pediatric patient populations, where feasible
- Use of simulation/modeling approaches to eliminate the need for additional clinical studies (e.g., in assessing drug-drug interactions)
- Patient-centric approaches to statistical analyses (i.e., intra-patient analysis of treatment effects) combined with efficient inter-group analyses against natural history or concurrent controls

Disease-based specific registries are urgently needed to promote and support broad research initiatives that will help in characterizing disease phenotypes, fostering understanding of disease pathology, and informing disease progression. These registries must incorporate processes and parameters to ensure patient identities are protected. Establishment of these non-proprietary registries requires close collaboration across sponsors, academia, and patient advocacy groups for optimal data sharing and data generation, and novel methods for data collection. Initiatives to encourage data sharing are needed since proprietary disease registries are often competitive and have nondisclosure stipulations. Future efforts should also aim to harmonize methodologies, establish standard guidelines for data extrapolation from source to target pediatric populations, and improve accessibility of approved drugs to patients. Establishing close collaboration and communication between the sponsor and regulatory bodies to address methodological and statistical challenges in real time during clinical development is key to streamlining the regulatory approval processes.

## Abbreviations

CLN2: Ceroid lipofuscinosis neuronal type 2
C-Path: Critical path institute
DCAs: Data contribution agreements
DUAs: Data use agreements
EIG: Efficient inter-group
FDA: Food and drug administration
ICH: International conference on harmonization of technical requirements for pharmaceuticals for human use
PK: Pharmacokinetic
QSP: Quantitative systems pharmacology
RDS: Randomized delayed start
RWE: Real-world evidence
RWE-RE: Real-world evidence-randomized enrichment
SOC: Standard of care
